# Gastric Disorders—From a Surgical Point of View

**Published:** 1914-05

**Authors:** Charles W. Hennington

**Affiliations:** Surgeon to the Rochester State Hospital; Assistant Surgeon, the Rochester General Hospital


// BUFFALO MEDICAL JOURNAL

Volume 69 MAY, 1914 No. 10

### ORIGINAL ARTICLES

### Gastric Disorders—From a Surgical Point of View

BY CHARLES W. HENNINGTON, B. S., M. D.
Surgeon to the Rochester State Hospital
Assistant Surgeon, the Rochester General Hospital

THIS subject of Gastric Disorders is a very fascinating one, because at present there is so much confusion and difference of opinion in the writings of those who should speak and write with authority. The whole subject seems to be undergoing a change. The term "disorder" is used advisedly because of its comprehensiveness and because least restricted to definite meaning. The remainder of the title, "from a surgical point of view," is to indicate the dominant trend of thought, yet the paper is not one of operative detail. By the title it is intended to lay emphasis on the great importance of the new surgical point of view and the influence which this has exerted upon the whole subecjt of Disorders of the Stomach,—how it has revolutionized entire chapters, how it is putting others to a severe test and how it has added entirely new chapters to the subject.

Historically this divergence of thought and opinion has, I think, a very interesting explanation. On reflecting a little it would seem as though the subject had passed through four very definite periods or stages or lines of development. They seem to me to be the following:

(1.) The earliest being the period of clinical observation of symptoms and signs, of possible causative factors and of deductions therefrom.

(2.) Then came the discoveries of gross and microscopic pathology and the more accurate conception of disease as a change in structure.

(3.) The great period of the introduction of the stomach tube and the analysis of test meals giving us a better knowledge of physiology of digestion and the knowledge of deranged function, or, in other words, pathological physiology of digestion.

(4.) Finally the period of more active surgical intervention and the comparison of symptoms with the exploration of the whole abdomen, giving us a more precise operative pathology, that is, a "pathology on the living."

These four periods or stages have succeeded each other in point of time both as to their beginnings and their gaining the ascendency and for a time predominating. They are, of course, not sharply limited from each other. They merge one into the other and still each retains its identity. In a way each continues and represents a point of view. Each can be looked upon as a progression in a given pathway along which much may still be hoped for and achieved.

The fact that these distinctions can be made, must not be taken to imply that we need ally ourselves with any one view point. We must be conservative and constructive. We must hold to that which has been definitely established and yet not fear to put it all to the test, even of this latest surgical criticism. That which is correct will remain.

Of our earliest view of "dyspepsia" based upon clinical observation, much in fact remains today, though it is seldom referred to in recent papers. All will acknowledge the abundance of gastric disorders due to dietetic causes or to noxious substances or habits such as alcohol, tobacco, etc. And we all likewise recognize a large class of stomach symptoms which are merely an expression of systemic derangements and general constitutional diseases, such as Bright's disease, tuberculosis, cirrhosis, cardiac disease, anæmia, etc.

Of the second or pathological period practically all remains, but our viewpoints have changed. We are no longer content with a terminal pathology. We must know disease in its earlier stages. By experimental pathology we want to discover, for instance, how ulcers begin and what are the factors of their causation.

The third period, that wonderful period of physiological study which added a new specialty to medicine, may be considered as beginning with the introduction of the stomach tube in 1869 by Kussmaul. By means of this instrument alone much could be discovered about the stomach, and it has made possible the analysis of test meals besides. Thus the motor and secretory functions of the stomach became a matter of study. We became acquainted with a large number of facts which are of diagnostic importance; and, in addition, there developed a great classification of all sorts of functional disorders.

The present surgical period has opened a controversy as to the accuracy of some of the facts of diagnostic import; and it has severely attacked much of the purely functional pathology of the gastro-enterologist.

In the first place it is beginning to be definitely conceded that in the past we have relied too much upon the so-called negative stomach analysis in cases in which cancer is suspected. The essential facts remain. In cancer total acidity is reduced definitely as a rule and free acid even more so, but we must not allow the absence of these findings to defer a diagnosis on other grounds or to oppose an exploratory operation. It is unnecessary to go into details as to whether these unsatisfactory examinations are due to the occurrence of cancer on old ulcers or otherwise.

Again, in gastric ulcer it was found that increased acidity was commonly present, but now we know that ulcer may be present regardless of these changes. In fact, their help in diagnosis of ulcer is slight.

That apparently thoroughly well established functional disorder of hyperchlorhydria has now lost its former position and is considerably discredited, due to the factors of causation discovered by the surgeon at abdominal exploration. Moynihan even states a view which may be extreme, namely, that "in *every* case chronic hyperchlorohydria means duodenal ulcer."

In addition, Moynihan, as representative of this new surgical point of view, has given us a distinctly new disease—"duodenal ulcer." He has supplied us with a clinical history of "pain two or three hours after eating and relieved by eating" as distinctive of a new clinical entity. The association of these symptoms with duodenal ulcer is a triumph of the new operative or living pathology. Operative pathology has also given us dilatation of the duodenum as described by Finney as another new disease entity.

Surgery has shown that an unfortunate source of delay in operating for gastric cancer has been due to the test meal, by depending upon and waiting for its late pathognomonic type. Pain and the presence of food remnants indicating obstruction at or near the pylorus are much earlier and much more important. Add to these a palpable tumor moving with respiration and the diagnosis is sufficiently probable to justify exploration. The study of bismuth meals by the Roentgen ray seems to be of the greatest value as evidence of obstruction. We should not wait for loss of weight and strength. The early diagnosis does not depend upon any sign or symptom due to the cancer itself, but on the mechanical conditions produced by the growth. The recognition of these mechanical conditions should be the aim of the diagnostician.

As to functional derangements, both surgery and radiography have directed attention to the relatively much greater importance of change in the motor activity of the stomach as compared with the secretory or sensory types. Thus a gastro-enterostomy probably gives relief mainly if not entirely by reason of drainage, that is by its influence on the motor function. But on the whole the surgical point of view is very critical of all these so-called pure functional disorders and of the entire classification and terminology of functional pathology based upon its main divisions of motor, secretory and sensory activity. Many of the secretory and sensory types have been referred to more precise anatomic diagnoses. Of the motor types some seem to be clinical entities. To clear up this matter, therefore, we will discuss the subject of "the surgery of the functional as well as organic disorders of the stomach."

At first thought it might seem that no matter how marked the degree of success attained by surgery in the case of organic disease of the stomach, the functional disorders were in a class of themselves and not open to surgical attack. In order that we may get a clearer conception of the designation "functional" in this connection, it seems wise to enter upon a definition or explanation. Of course, we need but mention that any organic disease of the stomach is accompanied by functional changes, either slight or marked. Also that various organic disorders in the immediate proximity of the stomach, such as adhesions, kinks, obstructions, and particularly inflammations, may be the causes of changes in the stomach itself and will make themselves evident by definite functional alterations. But in addition to these we have been learning of late that many so-called functional disorders of the anatomically normal stomach are reflex and have their physical basis in organic disease of other abdominal organs either near or remote, such as the duodenum, gall bladder, pancreas, appendix, small or large intestines. Our greatest recent advance has been the recognition of this fact, and it has impressed upon our minds the necessity of searching for and finding such diseases. Yet, when all is said, there remains, it seems, a smaller group of pure functional disorders of the stomach in the nature of pure neuroses of various types, more especially the motor, but, perhaps, to some extent also secretory and sensory. Of the motor variety there are hypermotility and spasm and motor insufficiency and atony. Whether these are open to surgical treatment is the question proposed.

Considered in an absolutely abstract and philosophic way, it must, of course, be granted that it is conceivable that even though the disorder be a pure neurosis, yet a surgical alteration of some sort might produce a favorable functional change and thus result in a relative or complete cure. The probabilities are that this field will be a limited one. From our present knowledge only those of a motor type of neurosis would seem to promise any reward.

We might, for instance, consider operation in a case of pylorospasm. The exploration might in the first place reveal an ulcer as the real cause of the pylorospasm. But even if the condition were a pure neurosis, as we have assumed, then an enlargement of the pylorus, as with the Finney pyloroplasty, would be of value. In congenital pylorospasm the same operation would seem to be indicated after failure with medical treatment.

Again, in some functional diseases, as motor insufficiency and atony of the stomach, certain surgical measures may be considered after suitable prolonged medical treatment has proven of no avail, and especially if special treatment has been directed against gastroptosis, which is often associated with this condition of motor insufficiency or atony. At operation an associated gastroptosis might be improved. But in this case, too, even if the condition were a pure neurosis, as we have assumed, then an operation, as the Finney pyloroplasty, to widen the pylorus would be of value. This would simplify the work of an atonic musculature. It should be remembered, too, that a functional disease of this kind may ultimately lead to considerable chronic dilatation of the stomach. Thus surgery might be employed in a functional disorder so as to prevent the occurrence of an organic condition.

It might appear from what has been said that the surgical viewpoint was unduly aggressive. If this were so it would be most unfortunate. However, from the correct point of view our diagnostic examination should be just as exhaustive as possible. We should use every procedure at our command. Not infrequently patients would willingly submit to all these measures. Often, as Bloodgood says, they get hundreds of dollars worth of treatment and not one cent's worth of real diagnosis. It would, however, seem unwise in this paper to go into detail as to history taking, physical and laboratory examination, test meals, etc., and radiographic examinations. Thereupon, in this field, a probable diagnosis may be sufficient justification to urge an exploratory operation.

But operations in chronic ailments are often undertaken too hurriedly, that is, not so much as to time, as too hurriedly in so far as to neglect to use those methods which we have at our disposal to determine the probable extent of the disease, and more particularly to determine the patient's resistance powers to operation. We should study the patient to determine his chances of recovery from the operation. This is the work of the medical man who is sufficiently skilled in the newer ways of determining the functional capacity of the heart and vascular system and of the kidneys. It may be that the new functional test as to the liver will ultimately be of service. We should know the operative capacity or vital resistance of our patients. A purely exploratory operation ought not to be undertaken unless we find on accurate clinical investigation that we are not taking undue chances.

*The Exploratory Operation.*—The most important question to be discussed in this connection is the degree to which the exploration should be carried out. This should be determined for each individual before the operation is begun. An exploration may be limited or so complete as to include an examination of the interior of the stomach. There are cases in which it would be entirely unwarranted to take the additional risk of opening the stomach even though the exploration is negative to this point. Whereas, in other cases it may be equally unwarranted to close the abdominal wound without having made the most thorough inspection of the lining of the stomach.

The exploration should, as a rule, be extended to all the other abdominal organs after simple inspection and palpation of the stomach has been done. The exploration should be especially thorough as to the duodenum, gall bladder and ducts, pancreas, lesser and greater omentum, appendix, large and small bowel with special reference to the regions of the sigmoid and the lower ileum. It is just the thoroughness of this exploration which will often change the diagnosis from a so-called functional disturbance of the stomach to a definite organic disease of some other abdominal organ.

An exploration should be systematic and thoroughly complete. It can be made a matter of considerable precision. It is a mental exercise, too, in so far as to rapidly recall the various lesions that should be looked for in each locality. Then, even if a definite lesion is discovered, the search should be continued so that no other lesion possibly of like or perhaps greater significance be overlooked.

Some points of special significance may be mentioned, for instance, in the presence of adhesions their cause should be definitely determined in each case. In the diagnosis it should always be stated to what primary cause the adhesions are attributable, for example, old ulcer, gall bladder infection, former inflammatory attacks, fœtal peritoneal folds, etc., etc. When one lesion as duodenal ulcer is found, the appendix should be examined with especial suspicion. Enlarged glands in the gastro-hepatic omentum speak as strongly for ulcer as for cancer and their location determines the exact area within which the disease is situated. Ulcers are overlooked for three reasons, either that we are not sufficiently acquainted with their characteristics, which is probably most often the case, or their inaccessible location, or that they are confined to the mucous membrane and do not involve the musculo-peritoneal coats until later. Nodular growths of the ovaries and pelvic glands both suggest a most thorough examination of the stomach.

Exploration of the interior of the stomach still calls forth considerable diversity of opinion as to its necessity and advisability. The matter must be determined in each individual case. As to the correct method there are several recommendations. It is essential that the patient be prepared for several days by proper attention to the mouth and throat and teeth and by stomach washing and emptying before the operation. Some suggest sterilized food, but fortunately most cooked food is comparatively sterile. Before making any opening into the stomach, it should, of course, be carefully packed off with gauze and withdrawn and lifted from the abdominal cavity as much as possible. The incision should be transverse, that is, at right angles to the axis of the stomach in order to avoid blood vessels. Coffey suggests that before making the incision several through and through stitches be taken on each side of the proposed line of incision. Traction is then made on these and the air in the stomach will rise to this point and so the incision can be made without spilling any contents. The fluid in the stomach can then be dipped out or aspirated with a bulb and rubber tube preferably by using that variety of stomach tube which has a bulb inserted in its course. Then the lining can be mopped dry with moist gauze. The examination may be by sight or palpation. The hand may be slipped into the lesser peritoneal cavity through a tear in the gastro-colic omentum, or a finger may be passed through the foramen of Winslow, and in this way various portions of the stomach may be brought into the incision. A speculum, such as a rectal speculum, can be passed through the incision to the pylorus and through into the duodenum if the diameter is one and one-half inches or less. Others depend upon palpation with the finger passing through the pylorus into the duodenum.

There are many other interesting details as to operative procedure which might be taken up. It has not been the intention, however, to speak on gastric surgery. I have merely attempted to give an exposition of the surgical point of view in approaching any case of gastric disorder.

(Read before the Rochester Pathological Society on October 16, 1913.)

---

Angina Pectoris With Transient Pericardial Friction Sound. Graham Steell, *Med. Chron.*, Manchester, November, 1913, reports four cases, three typic on angina, comprising his entire experience of 40 years' hospital practice. He has not found an allusion to the combination in text book but has found vague references in monographs.

